# Epochal changes and ethical issues, reflections on kidney transplantation

**DOI:** 10.1007/s40620-025-02350-1

**Published:** 2025-06-18

**Authors:** Jadranka Buturovic Ponikvar, Alejandra Orozco Guillen, Giorgina Barbara Piccoli

**Affiliations:** 1 Department of Nephrology, University Medical Centre Ljubljana, Ljubljana, Slovenia; 2 Department of Medicine, Faculty of Medicine, University of Ljubljana, Ljubljana, Slovenia; 3 Department of Medical Ethics, Faculty of Medicine, University of Ljubljana, Ljubljana, Slovenia; 4 Department of Nephrology, National Institute of Perinatology, Mexico City, Mexico; 5 Nephrology, Centre Hospitalier Le Mans, Le Mans, France

“The Future of Transplantation: Not a Privilege, but a Fundamental Right” is the title of an article which appeared in the June 2025 issue of *Transplantation* [1]. While the paper correctly points out the differences in costs entailed in dialysis and transplantation and the limited availability of both methods of kidney replacement therapy worldwide, it overlooks a number of issues whose analysis from an ethical standpoint could serve as the basis for further discussion. The article seems to imply that kidney transplantation is a cure, unlike dialysis, acknowledged as “an essential treatment for patients with kidney failure”, but described as “a palliative, not a curative measure”. This fails to take into account the important differences between cure and care, between treatment and palliation. A cure is normally seen as treatment that eliminates a disease or medical condition so that the patient is restored to health [2, 3]. Care is a process through which physical, psychological or social suffering is alleviated [2]. If the purpose of palliative treatment is to care for and not to cure a patient, i.e., if we are dealing with therapeutic interventions aimed at relieving symptoms and improving quality of life, rather than curing the underlying disease, then both kidney transplantation and dialysis can be considered palliative treatments. It should also be borne in mind that dialysis is a back-up for temporary or permanent transplanted kidney failure. The World Health Organization (WHO) defines palliative care as "an approach that improves the quality of life of patients and their families facing the problems associated with life-threatening illness, through the prevention and relief of suffering by means of early identification and impeccable assessment and treatment of pain and other problems, physical, psychosocial, and spiritual" [2]. This broad definition applies to most chronic diseases, even though the term generally refers to end-of-life care when the purpose of treatment (usually reduced in intensity) is no longer to prolong life but to help the patients live as comfortably as possible and make their death as peaceful as possible [3, 4]. The claim that the beneficiaries of dialysis are not the patients who receive treatment but the providers of machinery and supplies could be understood to mean that not only the medical industry but also dialysis physicians and nurses benefit from this situation [1]. The authors seem to have forgotten that the “uncomfortable reality— the global dialysis market, a multibillion dollar industry, thrives, whereas the field of organ transplantation struggles with funding, accessibility, and political support” does not account for the cruel lack of access to dialysis in many countries [1, 5]. With an estimated four million patients on dialysis, and an estimated demand that is twice as high, we see the patient’s fundamental right as access to care for kidney failure, not just and not necessarily transplantation [5]. As improvements in kidney transplantation have now made this treatment an option for elderly patients, the dialysis population in western countries has become older and is more likely to have severe or multiple comorbidities. It is this, not age per se, that limits the extent to which kidney transplantation can be prescribed; furthermore, kidney transplant waiting lists are long, far too long, at all ages [5, 6]. As stated in “The Future of Transplantation...” we lack organ donations, from both deceased and living donors; and we lack funds to create and maintain intensive care units, transplantation facilities and post-transplant care. Thus, even by increasing organ donation, given the current demographics of kidney failure, we are not going to empty the dialysis wards any time soon [6]. Kidney transplantation, in particular where a sound policy of expanded criteria is being applied, including elderly donors, donors with suboptimal kidney function, or donation after cardiocirculatory death, will probably not be a life-long treatment, especially for young patients [7]. In fact, considerable investments in procurement and in surgical and kidney preservation techniques are still needed. Are things so simple?—is it just a question of money? Transplantation has transformed many lives, offering hope where none existed. Yet as we champion its life-saving potential, we must also confront difficult questions. Framing access to transplantation only, and not to kidney replacement therapy as a whole, as a *"fundamental human right"* compels us to examine some of the ethical complexities this entails: reliance on living donors who assume significant risks, the undeniable reality that organ availability is often tied to loss of life, the morally fraught debates surrounding euthanasia coupled with organ donation, and above all, issues linked to organ shortage. Furthermore, this organ shortage has tragically given rise to unethical practices, including the exploitation of vulnerable populations, illicit organ trafficking, and the use of organs from executed prisoners, all forbidden by the Declaration of Istanbul.

Will xenotransplantation solve these problems? While we admire the heroic individuals that underwent the first xenografts, this does not appear to be a feasible universal solution, at least for several years to come [8].

**Fig. 1:**
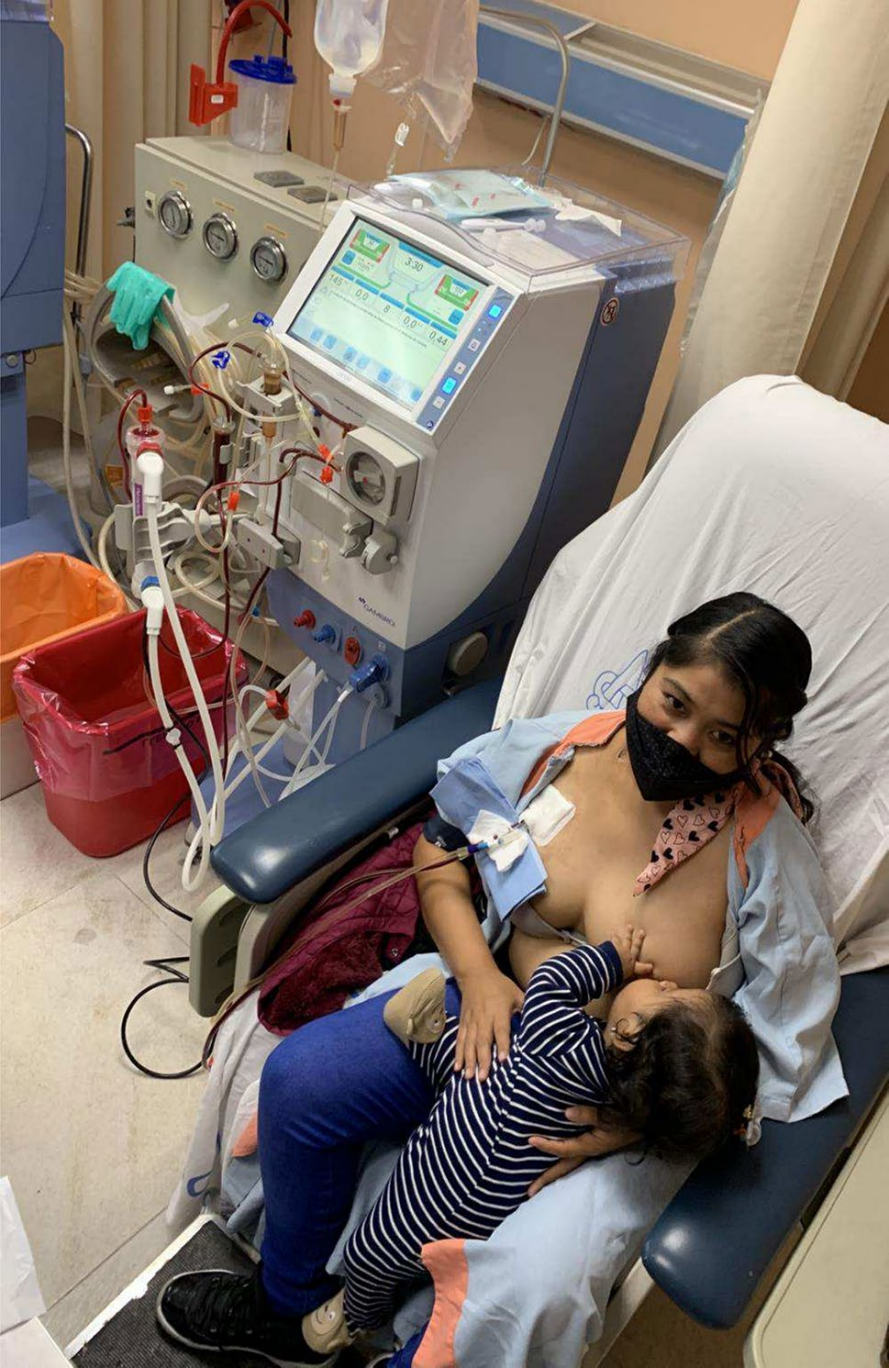
Dialysis is not "just" palliative care, but a bridge to even better care. In settings like Mexico, in which access to dialysis is still limited, and CKD is often diagnosed only in the late stages, the availability of a "good" dialysis treatment is vital. During a dialysis session, a young patient is breastfeeding her daughter who was born after she started chronic hemodialysis. Seventeen months after delivery she received a living kidney donor transplantation (donated by her sister); the image reminds us how dialysis and kidney transplantation should be integrated for the best patient outcomes. (Courtesy of AO, the patient agreed to share her images; cf also PMCID: PMC11843101 https://doi.org/10.1016/j.ekir.2024.11.021)

Our current challenge is not whether or not to support organ donation and transplantation—we must and we do—but rather to expand the right of all patients with kidney failure to receive the best treatment available, in line with the current clinical indications, without competition between kidney transplant and dialysis, and without demonizing dialysis, still needed and compatible with decades of fruitful life, and not just mere survival, a lesson of hope for all patients with kidney failure, whether or not they are on a kidney transplant list [9] (Fig. 1).
